# Genome Mining Reveals a Novel Nephthenol‐Producing Diterpene Synthase from the Sandfly *Lutzomyia Longipalpis*

**DOI:** 10.1002/cbic.202500292

**Published:** 2025-07-09

**Authors:** Charles Ducker, Catherine McKeown, Igor F. P. Da Silva, Isis Torres Souza, John A. Pickett, Antônio E. G. Santana, Neil J. Oldham

**Affiliations:** ^1^ School of Chemistry University of Nottingham University Park Nottingham NG7 2RD UK; ^2^ Institute of Chemistry and Biotechnology Federal University of Alagoas A. C. Simões Campus Maceió AL 57072‐970 Brazil; ^3^ Agricultural Sciences Centre Federal University of Alagoas Rio Largo‐AL 57100‐000 Brazil; ^4^ School of Chemistry Cardiff University Cardiff CF10 3AT UK

**Keywords:** biosyntheses, cryptic natural products, nephthenols, sand flies, terpene synthases

## Abstract

Populations of the sandfly, *Lutzomyia longipalpis*, use the diterpene sobralene as a sex/aggregation pheromone, which is likely produced in the insect through the activity of a recently discovered noncanonical terpene synthase (TPS). This study shows that the genome of this insect also contains another noncanonical TPS able to produce principally (*S*)‐(+)‐nephthenol (isoserratol) from geranylgeranyl diphosphate. This diterpene alcohol does not appear to be produced by the sandfly, nor is the corresponding TPS gene transcribed, hence these findings suggest that insects may be a promising source of TPSs for apparently cryptic terpenoid products.

## Graphical abstract

The discovery of a terpene synthase of insect origin that makes the apparently cryptic product nephthenol from geranylgeranyl diphosphate sheds light on hidden biosynthetic potential.

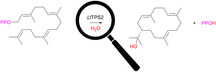

## 1 Introduction

**Scheme 1:**
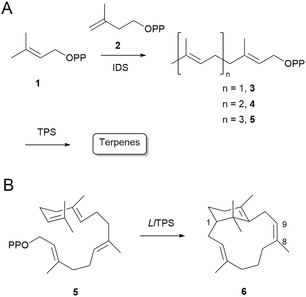
Biogenesis of terpenes. A) Biosynthesis of terpenes from DMAPP (**1**) and IPP (**2**) catalyzed by IDS and TPS enzymes. B) Production of the sandfly pheromone sobralene (**6**) from GGPP (**5**) by *Ll*TPS.

Terpenes and terpenoids are widely used for chemical communication in insects.^[^ ^1^ ^]^ Their biogenetic origin is the mevalonate (MVA) pathway, which leads to dimethylallyl diphosphate (DMAPP, **1**) and isopentenyl diphosphate (IPP, **2**). Coupling of these building blocks by isoprenyl diphosphate synthases (IDSs) to form geranyl, farnesyl, and geranylgeranyl diphosphate (GPP, **3**, FPP, **4,** and GGPP, **5**) provides precursors for terpenoid insect hormones, pheromones, and defensive secretions (**Scheme** **1**).^[^ ^2^ ^]^ Examples of hormones include the juvenile hormones,^[^ ^3^ ^]^ which control metamorphosis from the larval or nymphal stages to the adult form. Many instances of terpene/terpenoid insect pheromones are known, including (*E*)‐*β*‐farnesene in aphids,^[^ ^4^ ^]^ murgantiol in stink bugs,^[^ ^5^ ^]^ (6R,7S)‐himachala‐9,11‐diene in flea beetles,^[^ ^6^ ^]^ and a mixture of citral and geraniol in workers of the honeybee,^[^ ^7^ ^]^ to name but a few. Among terpenoid defensive secretions, iridoids (methylcyclopentanoids) are particularly common, being found in leaf beetle larvae, rove beetles, ants of the Dolichoderinae subfamily, and several species of stick insects.^[^ ^8^ ^]^

The sandfly *Lutzomyia longipalpis*, a vector of the *Leishmania* parasite, which causes the neglected tropical disease leishmaniasis, uses terpene pheromones as part of its mating strategy.^[^ ^9^ ^]^ Males release odorants to attract females and other males to lek mating sites. Different populations, or chemotypes, of *L. longipalpis* use different terpenes. Recently, we have identified a terpene synthase, which we termed *Ll*TPS, that produces the pheromone sobralene (**6**) from (*E,E,E*)‐GGPP (**5**).^[^ ^10^ ^]^ This novel diterpene is structurally related to the verticillenes but possesses a C8,C9‐(*Z*) double bond and hence both a positional and stereoisomeric rearrangement must occur during its biosynthesis (Scheme 1).^[^ ^11^ ^]^

The *L. longipalpis* genome contains numerous putative terpene synthases (TPSs) and IDSs, based on the presence of conserved amino acid motifs. Only a few of these candidates have been functionally characterized, with *Ll*TPS the only identified TPS.^[^ ^10^ ^]^ Here we report a new TPS from *L. longipalpis* that produces the diterpene alcohol nephthenol (isoserratol, **8**) from (*E,E,E*)‐GGPP (**5**).

## 2 Results and Discussion

**Figure 1:**
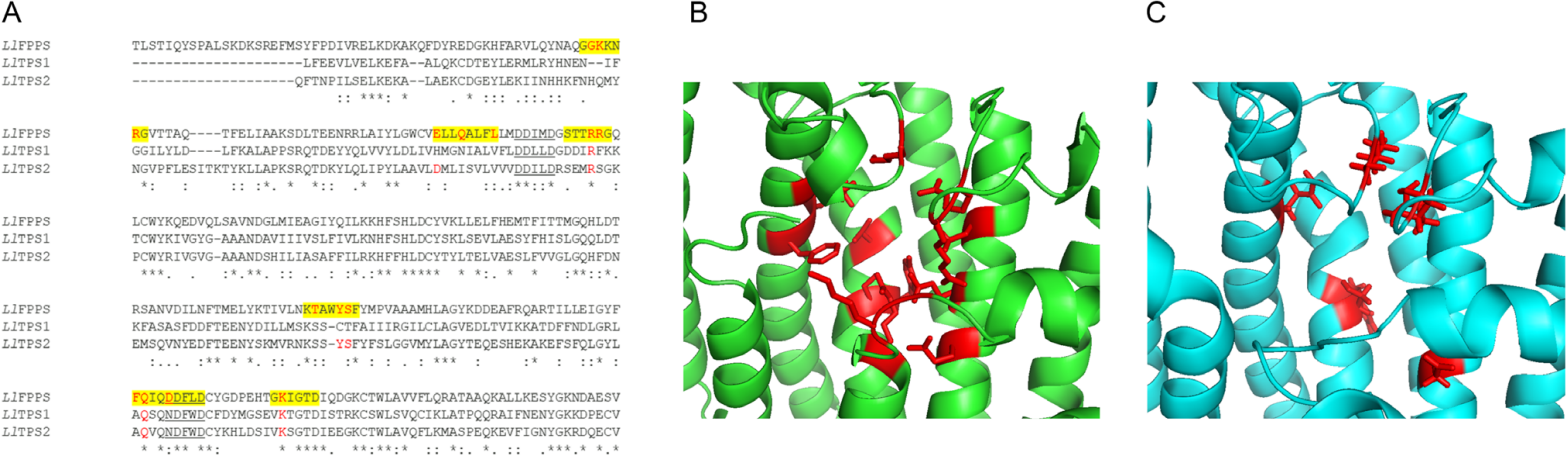
IPP‐binding motif substitutions in *Ll*TPS2. A) MUSCLE alignment of XP_055677521.1 (*Ll*FPPS, aa61‐356), XP_055691875.1 (*Ll*TPS1, aa10‐278), and XP_055692307.1 (*Ll*TPS2, aa13‐288). Underlined residues denote location of DDxxD motifs, with yellow highlighting predicted IBMs in *Ll*FPPS (IBMs 1–5 + SARM) and red identifying conserved residues reported to make direct contacts with IPP. B) AlphaFold model of *Ll*FPPS (rendered in PyMOL), showing predicted IPP binding residues (red). C) AlphaFold model of *Ll*TPS2, showing predicted IPP binding residues (red).

Protein BLAST searching of the published *L. longipalpis* genome^[^ ^12^ ^]^ against the *Ll*TPS sequence returned 25 hits for other FPPS‐like proteins (Figure S1, Supporting Information). Of these, the closest match was XP_055692307 (355 AAs), with 45% amino acid identity and 65% similarity (AAs 18‐343). The new sequence also possessed conserved first and second aspartate‐rich motifs (FARM and SARM), which are indicative of IDSs and TPSs.^[^ ^6^ ^]^ Employing the recently reported approach of Rebholz et al. 2023.^[^ ^1^ ^]^ to distinguish insect IDSs from noncanonical TPSs, using substitutions in the IPP‐binding motifs (IBMs), suggested the protein was a strong candidate for a TPS enzyme (**Figure** **1**). The sequence exhibited substitution of nine of the 15 normally conserved amino acids in predicted IPP binding regions (IBMs 1–5 and SARM), meaning that it was unlikely to bind incoming IPP co‐substrate efficiently and had potentially evolved from an IDS into a TPS. Following recombinant expression in *Escherichia coli*, the purified His‐tagged protein (see Experimental section and Figure S2, Supporting Information) was subjected to TPS activity profiling using a panel of isoprenyl diphosphate substrates.

**Figure 2:**
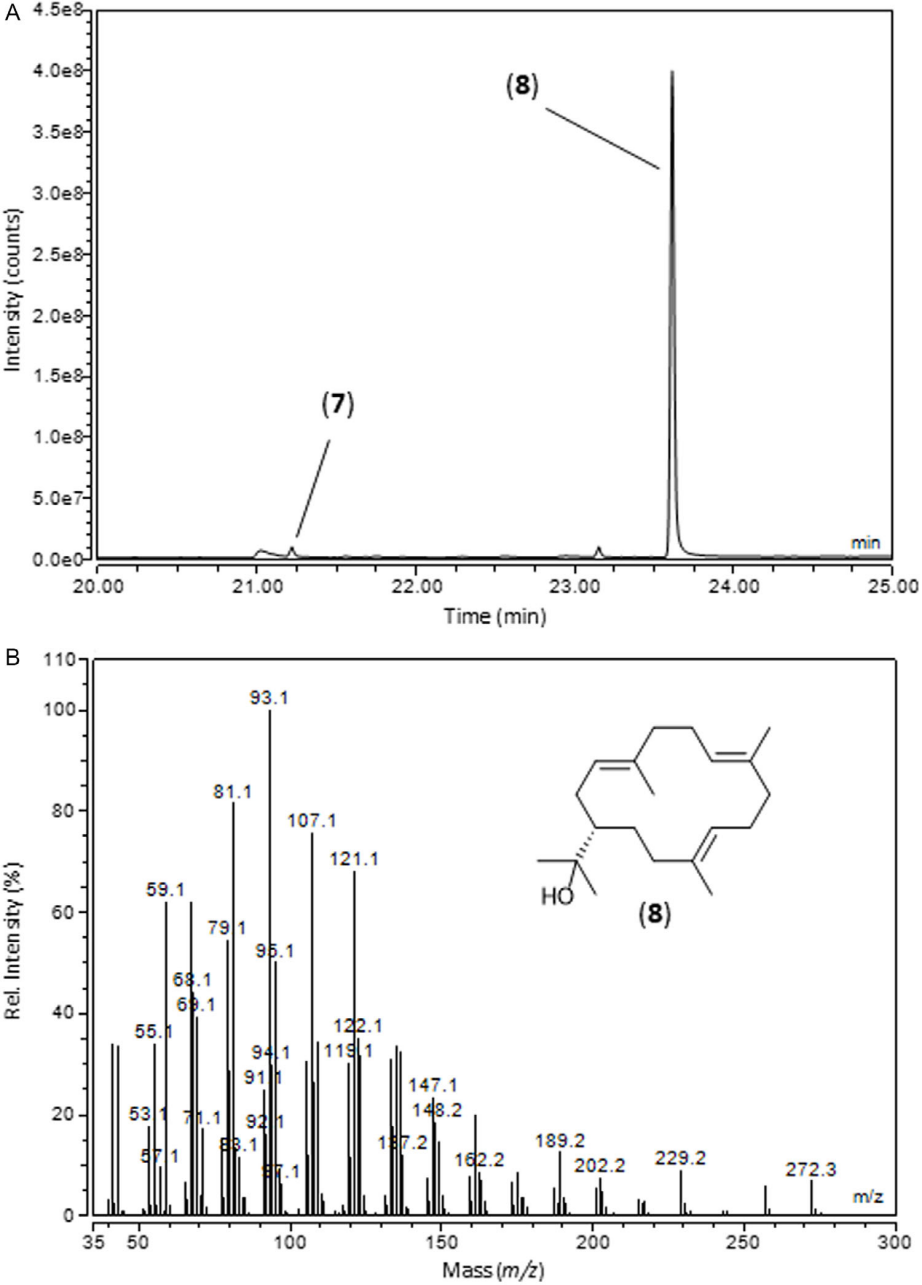
GC–MS analysis of the enzyme assay of *Ll*TPS2 with GGPP (**5**). A) GC showing the two products (unlabeled peaks are contaminants). B) EI mass spectrum of the major product.

Gas chromatography–mass spectrometry (GC–MS) analysis of pentane extracts from the enzyme assays revealed little or no TPS activity with the monoterpene precursors GPP (**3**) and its stereoisomer neryl diphosphate (NPP), or with the sesquiterpene precursors (*E,E*)‐, (*Z,E*)‐, and (*Z,Z*)‐FPP. Incubation with (*E,E,E*)‐GGPP (**5**), however, produced two diterpene products (**Figure** **2**). Based on this evidence, we propose that XP_055692307 is another *L. longipalpis* diterpene synthase, which we refer to hereafter as *Ll*TPS2, with *Ll*TPS (sobralene synthase) now becoming *Ll*TPS1. We cannot rule out that LlTPS2 might also exhibit sesterterpene synthase activity, although we found no evidence of geranyl farnesyl diphosphate production by IDS enzymes in *L. longipalpis*.^[^ ^10^, ^13^ ^]^ Examination of the electron ionization (EI) mass spectra of the two diterpene products of *Ll*TPS2 showed them to be similar to that of cembrene A (also known as neocembrene, **7**). The retention time and mass spectrum of the minor component were, in fact, identical to those of cembrene A, which was also a minor product of *Ll*TPS1 and present in the extract of *Boswellia occulta* (see below), confirming cembrene A as the minor product of *Ll*TPS2 (Figure S3, Supporting Information). The major component eluted significantly later than the diterpene hydrocarbon products of *Ll*TPS1. Determination of the retention index (DB‐5 stationary phase) for the major product of *Ll*TPS2 gave 2115, whilst retention indices of cembrene A, sobralene, and verticillene were 1955, 2009, and 2021, respectively. This led us to believe that the major product was a diterpene alcohol, rather than a hydrocarbon. Close examination of the EI‐MS showed a low‐intensity molecular ion at *m/z* 290 (Figure S4, Supporting Information), which supported this hypothesis; it being consistent with C_20_H_34_O. Additionally, a prominent ion at *m/z* 59 was present in the spectrum. This is not part of the regular terpene fragment ion series, and accurate mass measurement showed its formula to be C_3_H_7_O^+^ (measured *m/z* 59.047 ± 0.002, calc. for C_3_H_7_O^+^ 59.049), which was indicative of a 2‐hydroxypropanyl group. Taking the above data into account, we postulated that nephthenol (isoserratol, **8**) was the most likely candidate structure. An automated NIST library search of the spectrum reported cembrenol (serratol, **9**) as the top hit. The library spectrum included a relatively intense ion at *m/z* 59, which—whilst consistent with nephthenol (**8**)—seemed unlikely to be a major MS fragment of the serratol (**9**) structure. We therefore suspected an incorrect assignment in the NIST database, with the library spectrum actually being due to nephthenol (**8**).

Both nephthenol (**8**) and serratol (**9**) have been reported as components in the resin of *B. occulta*. *Boswellia* spp. are the source of frankincense, and the resin is rich in the diterpene alcohol incensole and its acetate. Ayubova et al. have fully characterized many of the products of *B. occulta* essential oil and have published authentic EI mass spectra of both nephthenol (**8**) and serratol (**9**).^[^ ^14^ ^]^ To confirm the identity of the major diterpene product from *Ll*TPS2 as nephthenol (**8**), we sourced a sample of *B. occulta* resin and purified both diterpene alcohols **8** and **9**.

By comparison of GC–MS data from the *Boswellia* products with those of the enzyme assay, it was clear that *Ll*TPS2 did, indeed, produce nephthenol (**8**) and not serratol (**9**) (Figure S5, Supporting Information). In order to identify the stereochemistry of nephthenol made by *Ll*TPS2, mg quantities of the diterpene were required for an optical rotation measurement. This was achieved by co‐transforming *E. coli* with plasmids containing i) genes for the lower MVA pathway and an FPPS (MVA → DMAPP, IPP → FPP) and ii) genes for *Ll*GGPPS and *Ll*TPS2, using the general approach of Morrone et al. and Martin et al.^[^ ^15^, ^16^ ^]^ Induction of these genes allows *E. coli* to generate diterpenes (such as nephthenol) from GGPP through TPS and IDS activity, following IPP and DMAPP production by the introduced MVA pathway. When accompanied by feeding with mevalonolactone, this strategy is reported to boost terpene yield in engineered *E. coli*. On a 100 mL‐scale culture, the method gave 2.8 mg of pure nephthenol, with ${\left[\alpha \right]}_{D}^{20}$ = + 21.7° (0.23, CHCl_3_), identifying the principal product as the (*S*)‐enantiomer, with an apparent *ee* of 55 %. (*S*)‐(+)‐Nepthenol is the enantiomer produced by octocoral *Eunicea* sp. and the TPS of a species of social amoeba (${\left[\alpha \right]}_{D}^{20}$ = + 45.5° (0.68, C_6_D_6_)),^[^ ^17^, ^18^ ^]^ whilst the (*R*)‐(‐)‐ form has been identified as a product of two *Streptomyces* TPSs and in the extracts of soft corals of the *Sinularia* genus (${\left[\alpha \right]}_{D}^{20}$ = ‐35.9° (1.0, MeOH)).^[^ ^19^, ^20^ ^]^ In addition to identifying stereochemistry, production of nephthenol from *Ll*TPS2 on the mg scale also afforded the advantage of allowing characterization by NMR (Figure S6, Supporting Information). Data were found to be identical to those reported for nephthenol by Rinkel et al.^[^ ^18^ ^]^ In contrast, the NMR data were not consistent with those of serratol (**9**) isolated from *B. occulta* (Figure S7, Supporting Information).

**Scheme 2:**
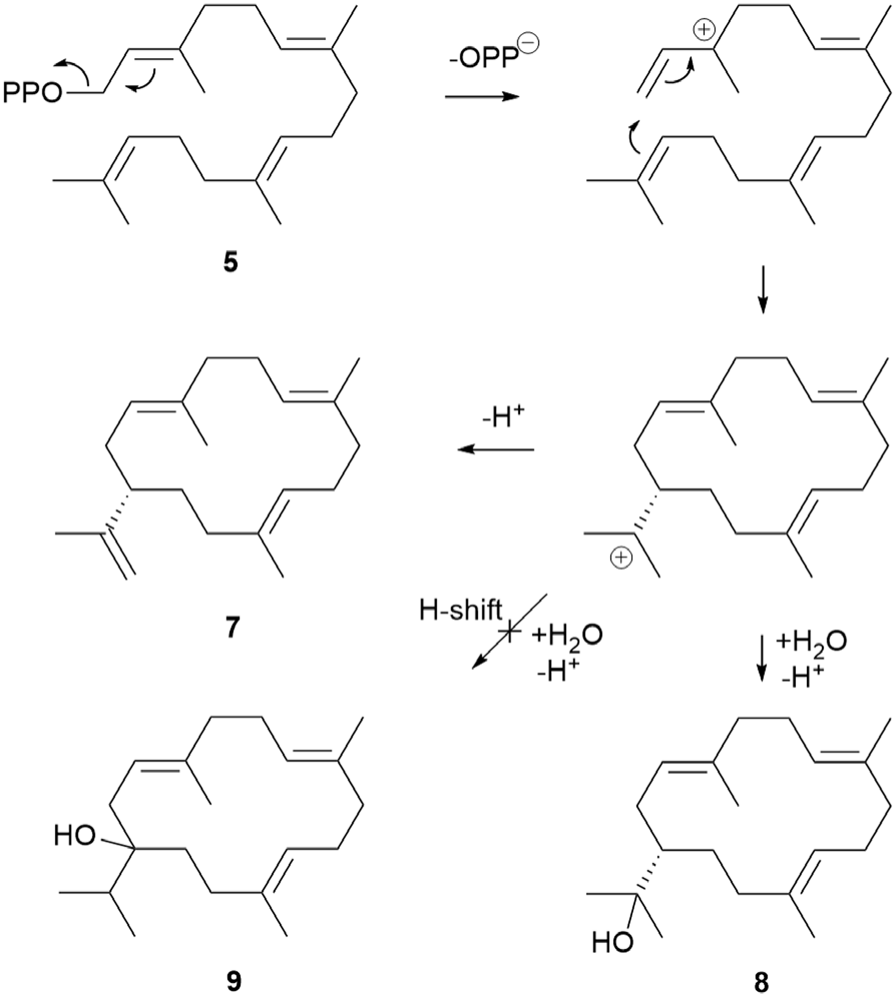
Mechanism for *Ll*TPS2‐catalyzed formation of cembrene A (**7**) and (*S*)‐(+)‐nephthenol (**8**) from GGPP (**5**). Their relationship to serratol (**9**), which is not seen as a product of *Ll*TPS2, is also shown.

To produce nephthenol (**8**), *Ll*TPS2 must first generate an allylic carbocation by removal of PPi from GGPP (**Scheme** **2**), which is catalyzed by Mg^2+^ ion(s) in the enzyme's active site. Cyclization to form the macrocyclic ring follows through attack by the C14,C15‐double bond at C1 to yield the cembrenyl cation. Direct quenching, either by the presence of a proximal ordered water molecule in the enzyme active site, or perhaps by exposure of the intermediate to bulk water, leads to nephthenol (**8**). E1 elimination of a proton from a methyl group adjacent to the cation gives cembrene A (**7**) (Scheme 2). Serratol (**9**), not seen as a product of *Ll*TPS2 would require a 1,2 hydride shift followed by quenching of the carbocation, now located on the ring. The first steps of nephthenol (**8**) production are identical to those for sobralene (**6**) but, in the case of sobralene (**6**), a second cyclization step to form the verticillenyl cation occurs in favor of quenching by water.

The presence of what may be described as a nephthenol synthase in *L. longipalpis* was unexpected, as—to the best of our knowledge—there is no record in the literature of this diterpene being a *L. longipalpis* product. Our findings reported here raise a number of possible explanations: either (*E,E,E*)‐GGPP (**5**) is not the native substrate of the enzyme, nephthenol (**8**) is not secreted by the exocrine glands of the insect, the alcohol is produced/utilized as a pheromone by an as yet unknown *L. longipalpis* chemotype, or *Ll*TPS2 is not functionally expressed in *L. longipalpis*.

Taking these possibilities in turn, (*E,E,E*)‐GGPP (**5**) proved to be the only meaningful substrate of *Ll*TPS2, with no activity shown towards GPP (**3**), NPP, or the FPP (**4**) stereoisomers. Moreover, the nephthenol (**8**) product was very pure by GC. Certain chemotypes of *L. longipalpis* are known to produce the homosesquiterpenes (*S*)‐9‐methylgermacrene‐B or (1*S*,3*S*,7*R*)‐3‐methyl‐*α*‐himachalene. Incubation of the enzyme with likely precursors of these homosesquiterpenes, (*E,E*)‐ or (*Z,E*)‐8‐methyl‐FPP, following a previously described approach,^[^ ^10^ ^]^ yielded no products and indicated that *Ll*TPS2 was not responsible for their synthesis.

The next possibility was that nephthenol (**8**) is not secreted by the exocrine glands of *L. longipalpis* but is a circulating metabolite of the insect, perhaps as a glycoside or other conjugate. Preliminary analyzes failed to detect the presence of such products, although a thorough metabolomic screen of the insect has yet to be undertaken. It is notable that some diterpene alcohols have antiprotozoal activity,^[^ ^21^ ^]^ and it is possible that nephthenol (or its derivatives) may be utilized by *L. longipalpis* in their interactions with the *Leishmania* parasite. Hoverflies of the genus *Microdon* have been reported to deposit nephthenol on their eggs during oviposition, perhaps as an antifungal agent.^[^ ^22^ ^]^ Studies on extracts of *L. longipalpis* eggs did not report the presence of this product, however.^[^ ^23^ ^]^

Regarding the suggestion that nephthenol (**8**) is the pheromone of an unknown *L. longipalpis* chemotype: this is a possibility, but many populations of this species of sandfly have been analyzed for their pheromone composition, due largely to the work of Hamilton and co‐workers.^[^ ^9^, ^24^, ^25^ ^]^ They and others have sampled sandflies (including, incidentally, other members of the *Lutzomyia* genus)^[^ ^26^, ^27^ ^]^ from many locations in Brazil and other parts of South and Central America, but have not, to our knowledge, found a nephthenol (**8**) producer. Assessment of male pheromone extract from Ceará, Brazil also found no evidence of nephthenol in this sandfly population.^[^ ^10^ ^]^

Whilst each of the above scenarios is possible, to us the most likely explanation was that *Ll*TPS2 is not constitutively expressed in adult *L. longipalpis* sandflies. Evidence for this view was provided by analysis of cDNA from insects collected in the field. This showed that whilst expressed *LlTPS1* could be clearly amplified from male cDNA (only), no similar result could be seen for *LlTPS2*, with either male or female cDNA (Figure S8, Supporting Information). This result indicated negligible transcription of the *LlTPS2* gene in the samples tested, the quality of which was controlled for using a housekeeping gene.^[^ ^29^ ^]^ We cannot, however, rule out that *Ll*TPS2 could be functionally expressed at a larval stage.

The evolutionary origin of TPSs in insects may also support the apparent low expression of *LlTPS2*. There is now a strong body of evidence showing that many insect TPSs have evolved from IDSs by amino acid substitution of IBM residues (see above).^[^ ^1^, ^6^ ^]^ This effectively removes IDS activity and allows the enzyme to evolve further into a TPS by active site modification. In the evolutionary journey to functional/utilized TPS it is likely that many inactive/unutilized sequences may be produced and that some of these may be retained in the genome. Indeed, *Nezara viridula* stink bugs possess a functional sesquipiperitol synthase despite no evidence of sesquipiperitol production within this species.^[^ ^28^ ^]^ The concept of silent genes, which possess the latent ability to code for the biosynthesis of cryptic natural products is a familiar one in the secondary metabolism of microorganisms^[^ ^29^ ^]^ but has not been widely explored in insects. We expect this situation will change as more insect species are sequenced. The recent discovery of canonical TPSs in insects provides further evidence for the complexity of terpene production in this class,^[^ ^30^ ^]^ but we note that the *L. longipalpis* genome does not appear to contain TPSs of the canonical type.

## 3 Conclusion

In summary, we report the identification of a new TPS from the sandfly *L. longipalpis*. (*E,E,E*)‐GGPP (**5**) appears to be the only active substrate, which is converted to the diterpene alcohol (*S*)‐(+)‐nephthenol (**8**), an apparently cryptic product of the adult sandfly.

## 4 Experimental Section

4.1

4.1.1

##### Materials and Methods

General: Purified water (18.2 MΩ) was generated using an ELGA Purelab system (ELGA, High Wycombe, UK). Organic solvents were sourced from Merck (Feltham, UK) unless otherwise stated. NMR spectroscopy was performed on a Bruker Avance III or a Bruker Avance III HD spectrometer (400 MHz ^1^H and 101 MHz ^13^C, Bruker, Billerica, MA) with chemical shift δ quoted in ppm, relative to residual solvent, and coupling constant *J* quoted in Hz. Optical rotation was measured on an ADP 440 polarimeter (Bellingham + Stanley, Weilheim, Germany).

##### Protein Expression and Purification

FPPS‐like homologs from the *L. longipalpis* genome were identified through BLASTp search using *Ll*TPS1 as the query. The closest match, XP_055692307 (*Ll*TPS2), was synthesized for bacterial expression in pET100/D‐TOPO (GeneArt, Thermo Fisher Scientific, Loughborough, UK) with an amino‐terminal hexahistidine tag. The expression vector was transformed into BL21(DE3)pLysS *E. coli*, and a single colony was used to inoculate a 20 mL LB culture for growth overnight at 37 °C with shaking at 200 rpm (100 μg mL ampicillin, 34 μg mL chloramphenicol). This was used the following morning to inoculate 1 liter LB, which was grown similarly until reaching OD_600_ 0.5, before protein expression was induced (0.75 mM IPTG) for 4 h at 30 °C. Cells were harvested by centrifugation (4000 rpm, 15 min, 4 °C) and pellets stored at −80 °C.

For purification, cell pellets were lysed in 35 mL wash buffer (50 mM Tris‐HCl pH 7.4, 100 mM NaCl, 10 % v/v glycerol, 20 mM imidazole and 1 mM DTT) supplemented with 1 % v/v Igepal CA‐630 and EDTA‐free cOmplete protease inhibitor (Roche, Welwyn Garden City, UK), and incubated at 4 °C for 15 min with mixing. Samples were then sonicated (12 × 10 sec) and centrifuged (14 000 rpm, 15 min, 4 °C), and prewashed nickel‐NTA agarose beads (0.5 mL bed) were incubated with the soluble fraction with mixing (2 h, 4 °C). Beads were washed with 3 × 10 mL wash buffer and protein was eluted with 3 mL wash buffer containing 400 mM imidazole. Protein was buffer exchanged into storage buffer (25 mM MOPSO pH 7.2, 100 mM NaCl, 10% v/v glycerol and 1 mM DTT) using PD‐10 columns (Cytiva, Little Chalfont, UK) and snap frozen (LN_2_). Proteins *Ll*FPPS and *Ps*IDS3 were expressed and purified similarly as described previously.^[^ ^13^ ^]^

##### TPS Activity Assays

Recombinant *Ll*TPS2 (2 μM) was incubated with either 50 μM GPP, FPP (*E,E* or *Z,E* or *Z,Z*) or (*E,E,E*)‐GGPP in TPS assay buffer (25 mM MOPSO pH 7.2 and 10 mM MgCl_2_, 100 μL volume) 30 °C, overlaid with 200 μL pentane. After 8 h the aqueous and organic layers were mixed with a glass Pasteur pipette, centrifuged (3000 × g, 2 min) and the aqueous layer was removed and discarded. The organic layer was evaporated to ≈50 μL under a nitrogen stream and 2 μL was submitted to GC–MS (see below). Dual IDS–TPS assays were carried out similarly, but with incubation of either *Ll*FPPS or *Ps*IDS3 protein (2 μM) with 100 μM 4‐methylGPP and 100 μM IPP (1 h, 30 °C), prior to addition of *Ll*TPS2 and pentane and overnight incubation as above. GPP (G6772), (*E,E*)‐FPP (44 722), and *(E,E,E*)‐GGPP (G6025) were purchased from Merck (Feltham, UK) and (*Z,E*)‐FPP (I‐0180), and (*Z,Z*)‐FPP (I‐0170) from Echelon Biosciences (Salt Lake City, Utah, USA). Synthesis of (±)‐4‐methylGPP has been described previously (Ducker et al. 2024).

##### Bacterial Production of Nephthenol

Nephthenol was produced using OverExpress C41(DE3) *E. coli* (Sigma‐Aldrich). The bacteria were transformed with two plasmids for recombinant protein production, which were as follows: 1) pMBIS (tetracycline resistant), encoding *ERG12*, *ERG8*, *MVD1* (all *Saccharomyces. cerevisiae*), *idi* (*E. coli*) and *ispA* (*E. coli*)(Addgene #17 817, Martin et al. 2003); and 2) pETDuet‐1 (ampicillin resistant), encoding *LlGGPPS* (*L. longipalpis*, subcloned into MCS2 through MfeI/XhoI sites), and *LlTPS2* (*L. longipalpis*, subcloned into MCS1 through NcoI/HindIII). Starter cultures were grown from a single colony overnight with shaking at 37 °C (10 mL LB, 100 μg mL ampicillin, 10 μg mL tetracycline, 0.1% w/v glucose, 200 rpm). A volume of this starter culture (2 mL) was then added to 100 mL TB media (12 g yeast extract, 24 g casein hydrolysate per liter, supplemented with 25 μg/mL carbenicillin and 10 μg mL tetracycline), and these were incubated at 37 °C and 200 rpm until reaching OD_600_ 0.6 (Morrone *et al.* 2010).^[^ ^15^ ^]^ The bacterial cells were induced with 1 mM IPTG and supplemented with 0.4% v/v glycerol and 70 mM potassium phosphate buffer, before incubation at 30 °C for 72 h at 200 rpm. Cultures were pulse fed with mevalonolactone at t = 0 h, t = 20 h and t = 28 h postinduction to a final concentration of 20 mM.

After incubation, the *E. coli* culture (100 mL) was removed from incubator, cooled to room temperature and pentane (50 mL) added. The biphasic mixture was vigorously stirred overnight at 5 °C. Following transfer to a separating funnel, a further volume of pentane was added (25 mL) and the layers allowed to separate. Ethanol (≈2 mL) was added dropwise to break up the emulsion. After 1 hr the aqueous layer was separated and extracted with a further volume of pentane (25 mL). The combined organic layers were washed with brine (25 mL) and dried over anhydrous sodium sulfate. Rotary evaporation of the solvent under vacuum yielded the crude product (15 mg). Nephthenol (**8**) was purified using silica gel chromatography (pentane:Et_2_O 4:1) to yield 2.8 mg of pure product. ${\left[\alpha \right]}_{D}^{20}$ = +21.7° (0.23, CHCl_3_); ^1^ H NMR (400 MHz, C_6_D_6_) δ 5.26 (tm, 1 H, *J* = 7.2), 5.15 (tm, 1 H, *J* = 7.2), 5.06 (tm, 1 H, *J* = 6.8), 2.25–2.0 (m, 11 H), 1.90 (dddd, 1 H, *J* = 7.3), 1.65 (m, 1 H), 1.58 (s, 3 H), 1.56 (s, 3 H), 1.53 (s, 3 H), 1.33 (m, 1 H), 1.23 (m, 1 H), 1.06 (s, 3 H), 1.05 (s, 3 H); ^13^C NMR (101 MHz, C_6_D_6_) δ 134.27, 133.04, 133.01, 126.93, 126.29, 125.33, 73.24, 48.77, 39.89, 39.31, 38.20, 29.02, 28.75, 27.86, 27.83, 25.15, 24.48, 15.78, 15.69, 15.44.

##### Extraction of B. Occulta Resin

Powdered Somalian *B. occulta* resin (26.9 g, The Incense Stick, Brooksville, FL) was extracted in cyclohexane (100 mL) with stirring for 1 h. The supernatant was decanted, and the remaining resin powder further extracted in cyclohexane (100 mL) with stirring overnight. The combined supernatants were filtered and the solvent evaporated to yield 12.2 g (41%) of crude extract. A portion of the extract (4.5 g) was dissolved in pentane (15 mL), assisted by ultrasound (1 min), and the solution removed from the remaining gum‐like solid. The pentane solution was purified using a silica gel (80 g) column using a solvent system of 3:1 pentane:diethyl ether. Fractions containing essentially pure serratol (**9**) were identified by GC–MS, combined, and rotary evaporated to yield serratol (108 mg). ^1^H NMR (400 MHz, CDCl_3_) δ 5.26 (tm, 1 H, *J* = 6.2), 5.02 (tm, 1 H, *J* = 7.1), 4.91 (tm, 1 H, *J* = 6.6), 2.31 (m, 1 H), 2.25–2.05 (m, 8 H), 1.95 (m, 2 H), 1.72 (m, 1 H), 1.68‐1.62 (m, 3 H), 1.60 (s, 3 H), 1.59 (s, 3 H), 1.57 (s, 3 H), 0.95 (t, 6 H). ^13^C NMR (101 MHz, CDCl_3_) δ 136.58, 135.55, 133.29, 125.94, 123.18, 120.91, 76.85, 39.91, 39.48, 34.93, 34.75, 34.55, 33.47, 24.82, 23.76, 16.81, 16.64, 16.38, 15.18, 15.06.

Fractions containing the majority of nephthenol (**8**) were contaminated with significant amounts of incensole, but one fraction containing a small quantity of essentially pure **8** was sufficient for GC–MS comparison with the product of *Ll*TPS2 (Figure S5, Supporting Information).

##### GC–MS

GC–MS analysis was carried out on either an Agilent 7890B GC equipped with a DB‐5 ms column (30 m × 0.25 mm × 0.25 μm, Agilent) coupled to a Jeol AccuTOF GCx system, or a Thermo ISQ 7000 GC–MS system equipped with a Restek Rtx‐1701 column (30 m × 0.25 mm × 0.25 μM). Both systems were operated in splitless mode, with the inlet temperature set to 200 °C. The Agilent GC oven was started at 50 °C with a 2‐min hold, before an increase of 15 °C min to 300 °C and a 1‐min hold. The helium carrier gas flow rate was set to 1 mL min. The Jeol MS was operated in positive EI mode, scanning from *m/z* 40–500 after a 4‐min solvent delay. For the Thermo GC–MS, the oven program started with a 3‐min hold at 35 °C, followed by an increase of 10 °C min to 260 °C and a 5‐min hold. The helium carrier gas flow rate was set to 1.5 mL/min. The MS was operated in positive EI mode, scanning from *m/z* 40–450 after a 5‐min solvent delay. Kovat's retention indices were estimated using an alkane standard (C_8_–C_20_, Merck, Feltham, UK). Product identities were confirmed by comparison of retention time and EI spectra against purified standards from Somalian *B. occulta* frankincense (The Incense Stick, Brooksville, FL).

##### Insect Collection

*L. longipalpis* flies of the (*S*)‐9‐methylgermacrene‐B‐producing 1 spot (1S) and sobralene‐producing 2 spot (2S) chemotypes were collected in the Alfa settlement (latitude −6.424 452 570, longitude −38.9 214 688), near Ico, Ceará, Brazil. Traps, of the CDC type, were placed near farms with chickens and goats from 5:00 pm to 6:00 am each night. Males and females were separated and placed (20 insects per vial) in RNAlater solution (ThermoFisher Scientific) (1 mL) to preserve genetic material.

##### RNA Extraction, cDNA Synthesis, and Polymerase Chain Reaction

RNAlater was removed from two separate vials of male and female *L. longipalpis* (a mixture of 1S and 2S types, 20 sandflies per vial), and the insects were resuspended in 350 μl RLT buffer (supplemented with 40 mM DTT) and transferred to Bel‐Art ProCulture Micro‐Tube homogenizers (SP Bel‐Art, Warminster, Pennsylvania, USA) for grinding with pestle. Homogenate was transferred to QIAshredder homogenizers (Qiagen, Hilden, Germany) and centrifuged (14 000 rpm, 2 mins), and total RNA was purified using RNeasy mini kit (Qiagen) as per manufacturer's instructions. Synthesis of cDNA was carried out by combining 1 μl 50 μM Oligo d(T)_20_ primer (Thermo Fisher Scientific), 1 μl 10 mM dNTPs and 280 ng template RNA in a total volume of 13 μl, before incubation at 65 °C for 5 mins. Added to this was a mixture of 5x SSIV buffer (4 μl), 100 mM DTT (1 μl), RNAseOUT^TM^ (1 μl, Thermo Fisher Scientific) and SuperScript IV Reverse Transcriptase (1 μl, Thermo Fisher Scientific), and samples were incubated at 50 °C for 10 mins and 80 °C for 10 mins.

PCR was carried out using Q5 High‐Fidelity DNA polymerase (New England Biolabs, Ipswich, Massachusetts, USA) as per manufacturer's instructions, using 1 μl male or female template cDNA per reaction. For amplification of *LlTPS1* and *LlTPS2*, cycling conditions were as follows: 98 °C for 30 secs; 30 cycles (98 °C for 10 secs, 61 °C for 30 secs, 72 °C for 35 secs); 72 °C for 2 mins. For amplification of housekeeper *LlEF1‐ α*, cycling conditions were as follows: 98 °C for 30 secs; 30 cycles (98 °C for 10 secs, 67 °C for 30 secs, 72 °C for 8 secs); 72 °C for 2 mins. Primers used were as follows: *LlTPS1* forward 5’ GTGAGAAGGTGGATTTCTG 3’, reverse 5’ CTAGAAGACCTAGAAGGATACC 3’; *Ll*TPS2 forward 5’ ATGGATAAGCAAACAGTTTACG 3’, reverse 5’ ATCTGTCATATTGTTGCCATC 3’; *LlEF1*‐ *α* forward 5’ GTGTCATCAAGGCTGTCAACTTC 3’, reverse 5’ TGGCTAGCTACTTCTTGGTCTTG 3’.^[^ ^31^ ^]^ PCR products were resolved on 1% w/v agarose and imaged on a Syngene NuGenius Plus gel imaging system (Syngene International Limited, India).

## Conflict of Interest

The authors declare no conflicts of interest.

## Supporting information

Supplementary Material

## Data Availability

Additional supporting data are provided in the ESI and are openly available from the University of Nottingham data repository at https://doi.org/10.17639/nott.7518.
