# Supplementary material for: Genome Mining Reveals a Novel Nephthenol‐Producing Diterpene Synthase from the Sandfly Lutzomyia Longipalpis
Source: Chembiochem. 2025 Jul 9;26(15):e202500292. doi: 10.1002/cbic.202500292 (PMC12376265; doi:10.1002/cbic.202500292)
Supplement: Supplementary file 1 — Supplementary Material [file CBIC-26-e202500292-s001.pdf]

Supporting information for:

Genome mining reveals a novel nephthenol-producing diterpene synthase from the sandfly *Lutzomyia longipalpis*

Charles Ducker, Catherine McKeown, Igor F. P. Da Silva, Isis Torres Souza, John A. Pickett, Antônio E. G. Santana and Neil J. Oldham*

Neil J. Oldham
Email: neil.oldham@nottingham.ac.uk

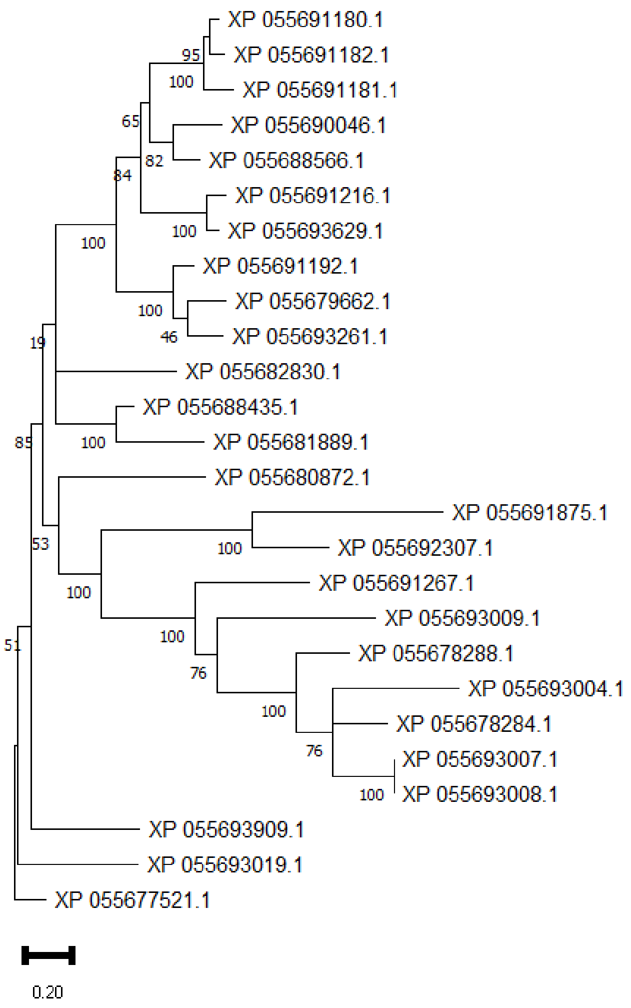

Figure S1. The *L. longipalpis* genome contains many FPPS-like genes. Phylogenetic tree of blast hits from XP_055691875.1 (*Ll*TPS1) against the *L. longipalpis* genome, rooted to XP_055677521.1 (*Ll*FPPS). All protein sequence hits were aligned using MUSCLE, with the maximum likelihood tree constructed in MEGA11 (LG+G+I, partial deletion of gaps), and phylogeny tested by bootstrap analysis (500 replicates).

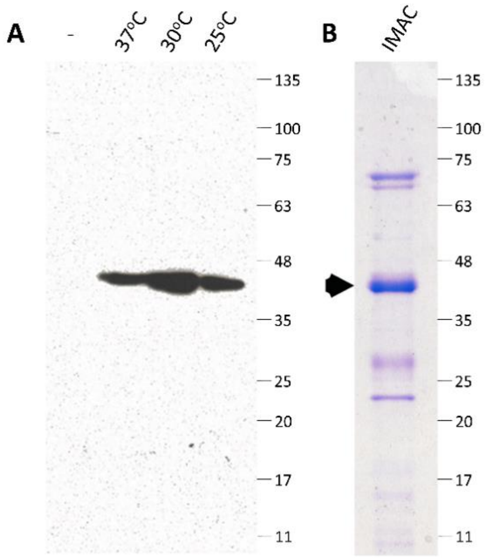

Figure S2. Recombinant expression and purification of *Ll*TPS2. (A) αHis immunoblot of lysate from BL21(DE3)pLysS *E. coli* harbouring pET100/D-TOPO-*Ll*TPS2 (N-terminally His-tagged). Bacterial cells were either left uninduced (-) or induced with 0.75 mM IPTG for 3 hours at 37 °C, 4 hours at 30 °C or 5 hours at 25 °C (30 °C was chosen for protein purification). (B) Recombinant *Ll*TPS2 (1.5 μg) following purification using immobilised metal affinity chromatography (IMAC) resolved by SDS-PAGE (5-20% gradient) and stained using Coomassie Blue R-250. Numbers to right denote MW markers (kDa). Protein is identified by arrowhead.

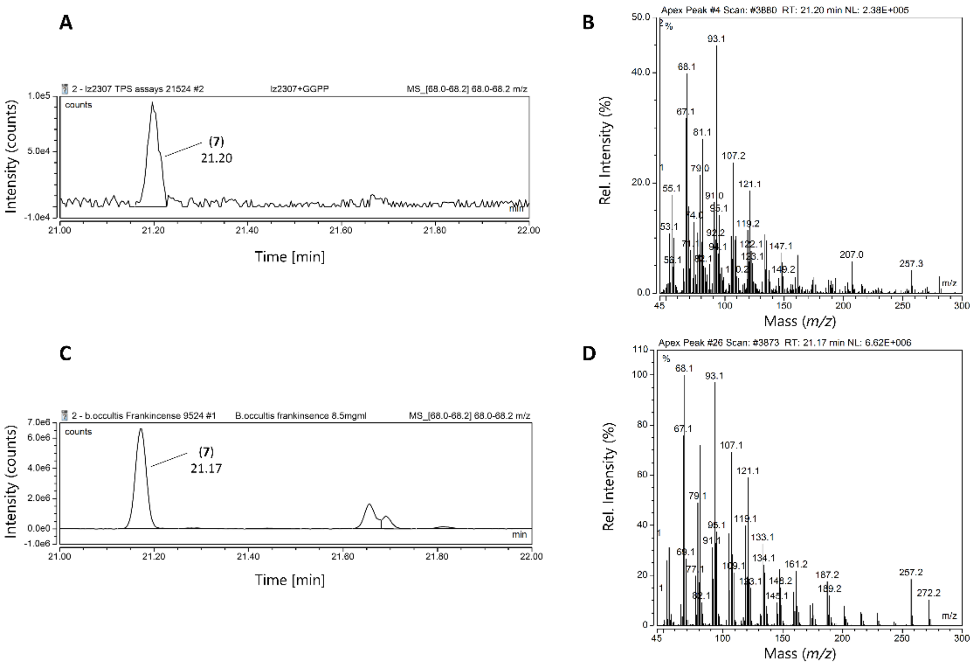

Figure S3. Cembrene A is a minor product of *Ll*TPS2. (A) GC-MS chromatogram of the enzyme assay of *Ll*TPS2 with GGPP (EIC *m/z* 68.1). (B) EI mass spectrum of cembrene A (**7**) peak from chromatogram (A). (C) GC-MS chromatogram of *B. occulta* frankincense extract (EIC *m/z* 68.1). (D) EI mass spectrum of cembrene A (**7**) peak from chromatogram (C). A Restek Rtx-1701 GC column was used in both cases.

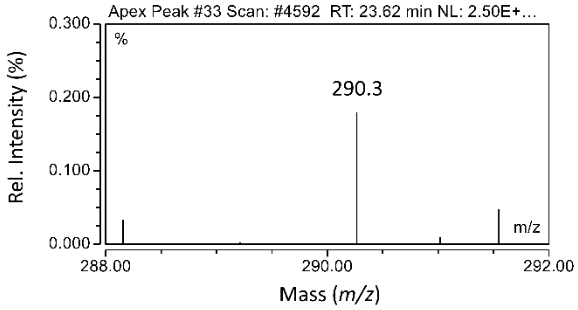

Figure S4. Major product of *Ll*TPS2 displays a molecular ion at *m/z* 290, consistent with a diterpene alcohol. Zoomed in EI spectrum from Figure 2.

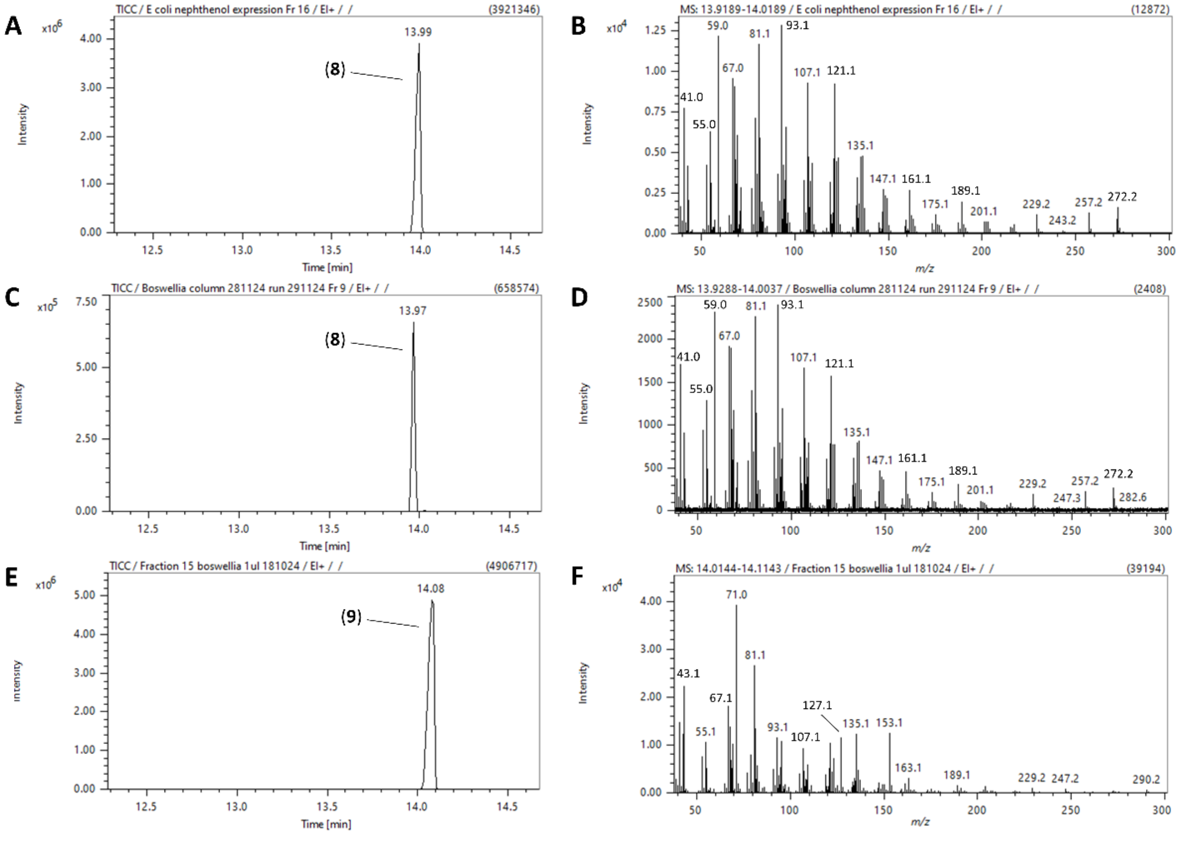

Figure S5. Nephthenol is the major product of *Ll*TPS2. (A) GC chromatogram of purified nephthenol (**8**) produced in *E. coli* by *Ll*TPS2. (B) EI mass spectrum of peak from chromatogram (A). (C) GC chromatogram of nephthenol (**8**) purified from *B. occulta* frankincense extract. (D) EI mass spectrum of peak from chromatogram (C). (E) GC chromatogram of serratol (**9**) purified from *B. occulta* frankincense extract. (F) EI mass spectrum of peak from chromatogram (E). A non-polar DB-5 GC column was used in all cases.

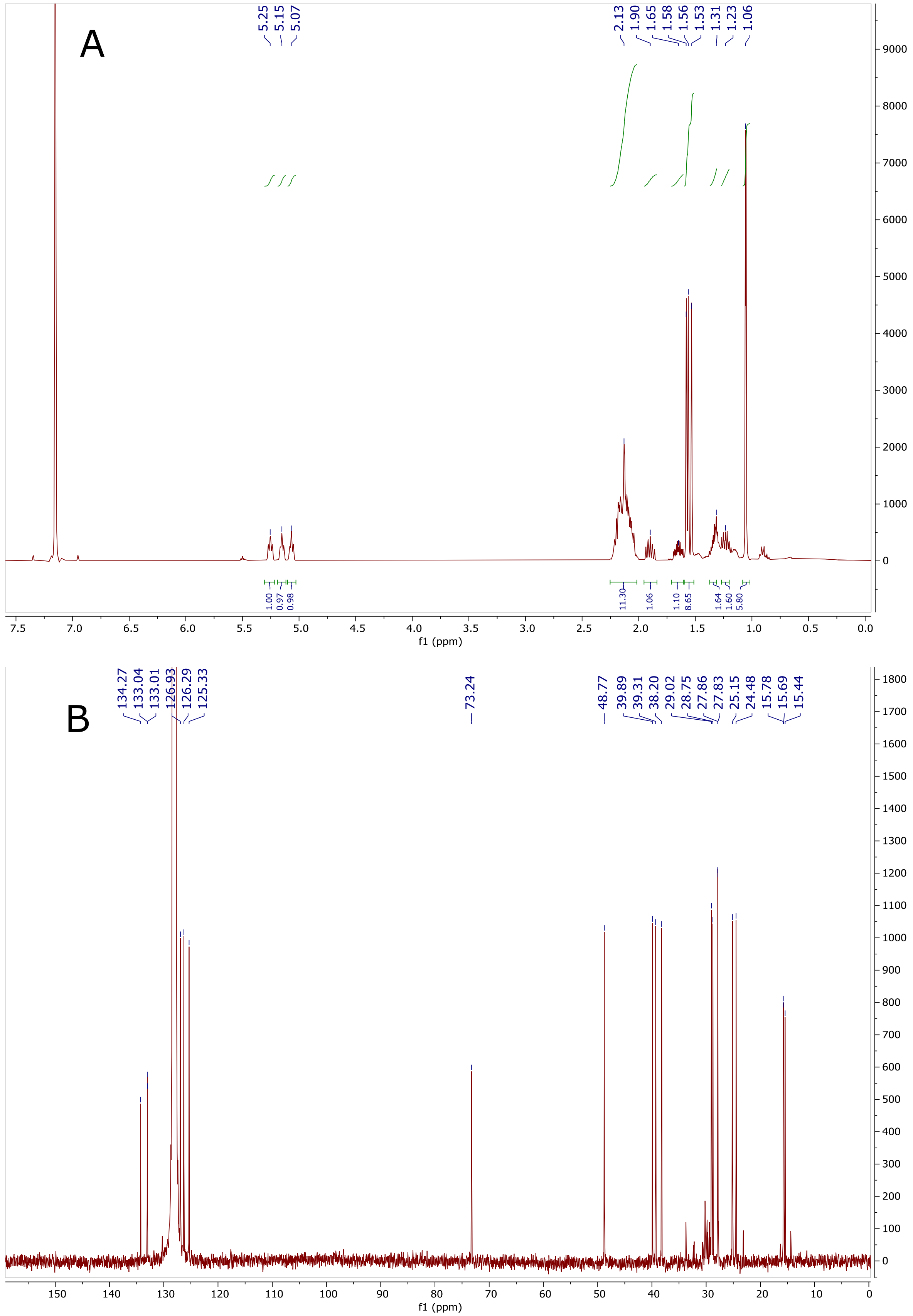

Figure S6. ^{1}H NMR (A) and ^{13}C NMR (B) spectra (in C_6D_6) of nephthenol (**8**) produced in *E. coli* by *Ll*TPS2.

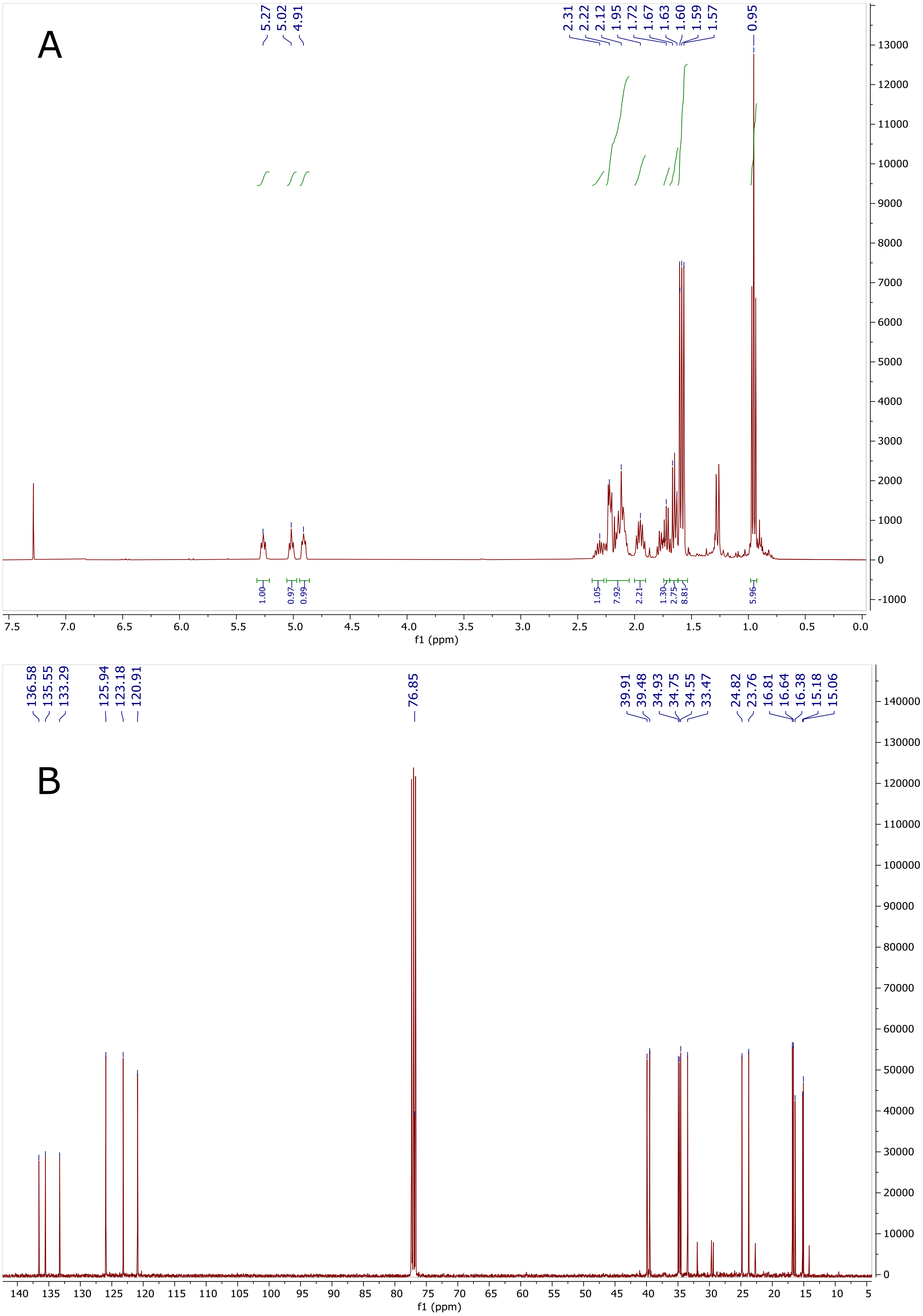

Figure S7. ^{1}H NMR (A) and ^{13}C NMR (B) spectra (in $CDCl_3$) of serratol (**9**) from *B. occulta*.

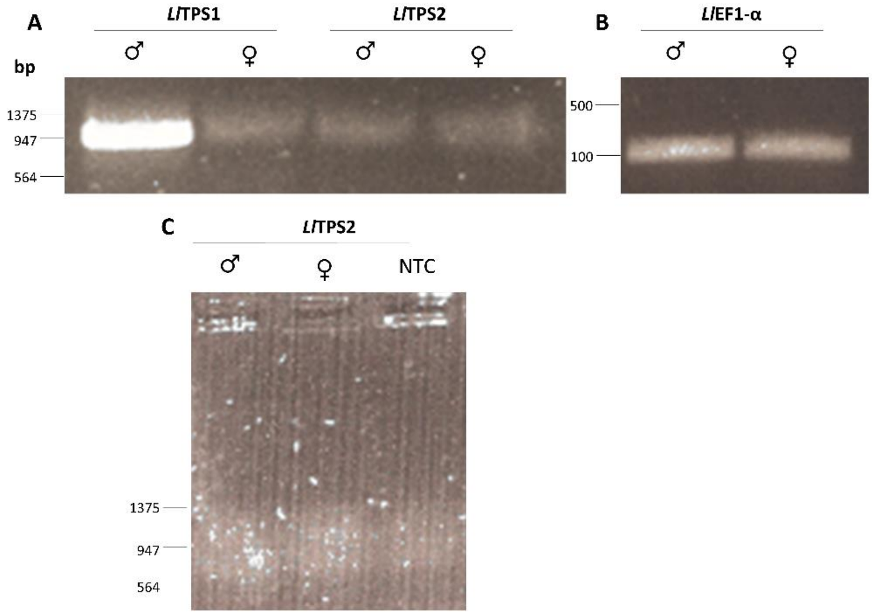

Figure S8. *Ll*TPS2 is not expressed in *L. longipalpis* adults. (A) PCR products from cDNA generated from male (♂) and female (♀) sandflies obtained near Ico, Ceará, Brazil showing presence of transcribed *LlTPS1* (1137 bp) in males (only) and essentially no transcription from *LlTPS2* gene (1062 bp) in either males or females. (B) The positive control gene, *LlEF1-α*, is transcribed in both males and females (93 bp fragment), indicating a similar quality of cDNA from each sex. (C) Confirmation of no evidence of *LlTPS2* expression compared to background level in no template control (NTC).
